# Editorial: Current advances in multimodal human brain imaging and analysis across the lifespan: From mapping to state prediction

**DOI:** 10.3389/fnins.2023.1153035

**Published:** 2023-02-13

**Authors:** Layla Banihashemi, Jinglei Lv, Minjie Wu, Liang Zhan

**Affiliations:** ^1^Department of Psychiatry, University of Pittsburgh, Pittsburgh, PA, United States; ^2^School of Biomedical Engineering and Brain and Mind Centre, University of Sydney, Camperdown, NSW, Australia; ^3^Department of Electrical and Computer Engineering, University of Pittsburgh, Pittsburgh, PA, United States

**Keywords:** multimodal neuroimaging, functional MRI (fMRI), resting-state connectivity, diffusion-weighted imaging (DWI), PET, EEG, MRI methodologies

In preclinical animal models, researchers can, within the same thin slice of tissue, probe activity within neurons [e.g., immediate early gene protein products (Mcreynolds et al., 2018; Aparicio et al., 2022)], examine neurons' projections and/or synaptic innervations [e.g., tract or viral tracing (Card and Enquist, 1999; Saleeba et al., 2019)] and determine neurochemical phenotypes [e.g., immunohistochemistry (Magaki et al., 2019)]. Great mechanistic specificity can be achieved with preclinical approaches. In understanding the human brain, neuroimaging affords researchers the opportunity to noninvasively probe brain structure, function and connectivity, but is not without limitations. For example, the blood oxygen level-dependent (BOLD) signal in functional magnetic resonance imaging (fMRI) is a proxy for neural activation based on the displacement of deoxygenated by oxygenated hemoglobin and is not, itself, neural activity (Huettel et al., 2009). Further, diffusion-weighted imaging (DWI) and derived tractography provide inferences of white matter structure based on the diffusion of water molecules restricted by neural components and do not represent specific neuronal targets or synaptic innervations. Thus, interpretation of neuroimaging findings is greatly enhanced by known neuroanatomical and functional literature from preclinical models, and efforts to find convergence across these approaches are highly important (e.g., Folloni et al., 2019; Haber et al., 2021). Similarly, a consensus between anatomical techniques in preclinical models or post-mortem human brain (e.g., blunt and/or fiber dissection) and neuroimaging (e.g., tractography) is also significant (Wu et al., 2016; Oler et al., 2017; Pascalau et al., 2018).

Despite limitations of neuroimaging, there is great potential to leverage the advantages of, and integrate distinct neuroimaging modalities to achieve a broader picture of neural dynamics and a greater mechanistic understanding of myriad developmental, affective, cognitive and clinical issues. Distinct neuroimaging modalities may reveal relationships with different dimensions of early experience providing insights into neurodevelopment. For example, diffusion spectrum imaging revealed opposing relationships of childhood threat (i.e., abuse and traumatic events) and deprivation (i.e., socioeconomic) on stria terminalis white matter (Banihashemi et al., 2021b). Further, resting-state functional connectivity revealed relationships between traumatic events and central visceral network connectivity (Banihashemi et al., 2022), while stressor-evoked activity revealed relationships primarily with childhood socioeconomic deprivation (Banihashemi et al., 2021a). Clinically, multimodal neuroimaging can perhaps provide earlier or more accurate detection of psychopathology (Lei et al., 2020; Vai et al., 2020).

Brain structure (e.g., white matter microstructure) and function (e.g., resting-state functional connectivity) have a reciprocal relationship that facilitates global and integrative brain processes (Sporns et al., 2000, 2004; Honey et al., 2007; Zhu et al., 2014; Lv et al., 2023). Thus, multimodal neuroimaging research can better capture integrated neural mechanisms underlying complex processes. New resources, tools and methods have been developed to examine multimodal neuroimaging data (Paquola et al., 2021; Cruces et al., 2022; Fortel et al., 2022). For instance, Nozais et al. examined the joint contribution of white and gray matter to large-scale resting-state networks elucidating the structural interconnections and pathways of communication that yield functional connectivity at rest (Nozais et al., 2022).

In this current Research Topic, authors demonstrated the breadth of how multimodal neuroimaging can surpass unimodal neuroimaging across developmental, clinical and methodological domains. Zhang et al. addressed neurodevelopment as it relates to the preterm infant brain by using a novel fusion framework combining functional and structural data. They integrated canonical correlation analysis and locality preserving projection to examine relationships between multimodal connections (fMRI and DWI) and found connection features that distinguish preterm and term-born infants. Their approach revealed novel insights into the global manner in which preterm birth impacts neurodevelopment, identifying local intra-network functional connections and long-range inter-network structural connections that differentiate between pre-term and term-born infant brains.

Zhu et al. presented new methodology featuring brain network construction under a unified framework of joint fMRI and DWI (i.e., functional and structural connectivity). Their method considered relationships between multiple brain regions and a PageRank algorithm that extracts significant node information from the unified network. Zhu et al. applied their method to a clinical problem—classifying epilepsy diagnoses, comparing normal controls (NC), frontal lobe epilepsy (FLE) and temporal lobe epilepsy (TLE). Their methods achieved the highest classification accuracy compared to unimodal methods when classifying against NC and achieved among the highest accuracies when classifying FLE v. TLE.

Tang et al. developed a new interpretable hierarchical graph representation learning framework for brain network regression analysis using multimodal MRI data. They used this approach to predict a variety of affective, somatic, cognitive and behavioral measures and found that the proposed framework achieved the best performance compared to baseline methods; this was attributed to extraction of graph local structures as low-level features and preservation of these into high-level space hierarchically.

Sun et al. developed new methods that capitalize on synergies between PET and DWI data, using DWI-derived structural connectivity and PET intensity to denoise PET images. Their CONNectome-based Non-Local Means (CONN-NLM) filter provides more informative denoising by weighting similar-intensity PET voxels and highly connected voxels more heavily. This yielded greater PET image quality and lesion contrasts and produced superior denoising effects compared to filters not utilizing DWI data.

Finally, Babaeeghazvini et al. reviewed the convergence of structure and function with associations between white matter microstructure and electro-encephalography (EEG). They note that white matter microstructure may influence the velocity of communication between brain regions and across hemispheres, and that amplitudes and latencies of event-related potential components may reflect pathological differences in structure; yet, the diversity of findings calls for more standardization of EEG analysis.

To conclude, the field would benefit significantly from effective use of multimodal approaches in the methods development space and in basic, clinical and translational research. This requires open science and enhanced accessibility of tools that process and analyze multimodal neuroimaging data. Future directions can include enhanced integration of “neurochemical” imaging modalities (e.g., MR-spectroscopy) with structural and functional modalities. The reports highlighted in this topic are an excellent demonstration of how multimodal approaches can improve methodologies, predictive power and clinical classification abilities to ultimately identify neural markers of psychopathology risk and guide more targeted treatments.

## Author contributions

All authors listed have made a substantial, direct, and intellectual contribution to the work and approved it for publication.
